# Considerations when using nutrient inventories to prioritize water quality improvement efforts across the US

**DOI:** 10.1088/2515-7620/abf296

**Published:** 2021-04-16

**Authors:** Robert D Sabo, Christopher M Clark, Jana E Compton

**Affiliations:** 1 Center for Public Health and Environmental Assessment, Office of Research and Development, U.S. Environmental Protection Agency, Washington, D.C., United States of America; 2 Center for Public Health and Environmental Assessment, Office of Research and Development, U.S. Environmental Protection Agency, Corvallis, OR, United States of America

**Keywords:** nitrogen, phosphorus, water quality, nutrients, watershed, inventory

## Abstract

Ongoing water quality degradation tied to nitrogen and phosphorus pollution results in significant economic damages by diminishing the recreational value of surface water and compromising fisheries. Progress in decreasing nitrogen and phosphorus pollution to surface water over the past two decades has been slow. Limited resources need to be leveraged efficiently and effectively to prioritize watersheds for restoration. Leveraging recent nitrogen and phosphorus inventories for the years 2002, 2007, and 2012, we extracted relevant flux and demand terms to help identify US subbasins that are likely contributing a disproportionate amount of point and non-point source nutrient pollution to surface water by exploring the mean spatial distribution of terrestrial anthropogenic surplus, agricultural surplus, agricultural nutrient use efficiency, and point source loads. A small proportion of the landscape, <25% of subbasin area of the United States, contains 50% of anthropogenic and agriculture nitrogen and phosphorus surplus while only 2% of landscape contributes >50% of point source loads into surface water. Point source loads are mainly concentrated in urban areas across the country with point source loading rates often exceeding >10.0 kg N ha^−1^ yr^−1^ and >1.0 kg P ha^−1^ yr^−1^. However, the ability for future upgrades to wastewater treatment plant infrastructure alone is unlikely to drive further improvement in water quality, outside of local water ways, since point source loads only account for ~4% of anthropogenic N and P surplus. As such, further progress in boosting nutrient use efficiency in agricultural production, usually lowest in areas of intensive livestock production, would likely contribute to the biggest gains to water quality restoration goals. This analysis and the corresponding database integrate multiple streams of information to highlight areas where N and P are being managed inefficiently to give decision makers a succinct platform to identify likely areas and sources of water quality degradation.

## Introduction

Recent calls to rapidly improve nutrient use efficiency associated with food, fiber, and fuel production (Houlton *et al* 2019), decrease emissions of nitrogen oxides and ammonia from the combustion of fossil fuels and agriculture (Li *et al* 2016), and constrain the unintentional release of nitrogen and phosphorus flows from waste management systems can greatly mitigate the globally, well-documented negative effects of excess nutrients on water quality (Galloway *et al* 2003, Billen *et al* 2013). Over the past four decades, complex combinations of technological innovations, educational outreach, regulatory enforcement, economic drivers, and voluntary approaches have led to substantial progress in decreasing NO_x_ emissions from power plants and vehicles (e.g, (Lloret and Valiela 2016, Sullivan *et al* 2018)), reducing point source loads into surface water (Ivahnenko 2017) and increasing nutrient use efficiency in agriculture throughout Europe and North America ( Zhang *et al* 2015). Despite these positive developments, the reduction of nitrogen and phosphorus pollution to surface waters over the past two decades has been slow (Oelsner *et al* 2017). Sales bans on phosphate-containing detergents (Sabo *et al* 2021), declines in atmospheric N deposition (Eshleman and Sabo 2016), and costly efforts to upgrade wastewater treatment plants have had a positive, though region-specific influence on water quality trends (Ator *et al* 2019, Fanelli *et al* 2019). However, these sources of nitrogen and phosphorus, generally managed through various regulatory mechanisms, usually make up a relatively smaller fraction of nutrient inputs to larger basins in turn limiting their potential to bring about broader improvements in water quality (Houlton *et al* 2013, Tian *et al* 2020). Understanding the likely location of major sources of nutrient pollution across the country can inform prioritization of watershed restoration efforts, whether planning at the watershed, state, or national level to better prioritize scarce resources earmarked for water quality restoration (Sobota *et al* 2013, Roy *et al* 2021).

Advances in the development of standardized, spatially explicit nitrogen and phosphorus inventories for the conterminous US offer an unprecedented opportunity to help inform the prioritization of watersheds for potential restoration efforts at the local, state, and national level (Sabo *et al* 2019, Zhang *et al* 2020). The ability to identify hot spots of likely point and non-point source pollution across the landscape as well as identify inefficiencies in the use and handling of nitrogen and phosphorus in both agricultural and urban settings also allow decision makers and stakeholders to develop targeted, locally tailored strategies to maximize downstream water quality improvements within larger basins (Sobota *et al* 2013, Swaney *et al* 2018, Swaney and Howarth 2019). Different industries within catchments have different potentials for mitigating nutrient pollution as well as costs. Some examples include tertiary treatment for wastewater treatment plants (Cohen and Keiser 2017), scrubbers for emission stacks, buffer strips and wetland restoration for row crops (Weller *et al* 2011, Weller and Baker 2014), and improved lagoon management for concentrated animal feeding operations (CAFOs) (Schröder *et al* 2011, Sobota *et al* 2013). Prioritizing action is not as simple as looking for the largest contributor, as many areas are mixed use and thus pollution sources may co-dominate. Furthermore, in crop dominated areas it is not as simple as calculating fertilizer input rates, as areas vary in their ability to produce more biomass, thus, the remaining surplus nutrients will vary across the landscape, even for regions with similar land cover and fertilizer application rates (Lu *et al* 2019, Swaney and Howarth 2019). Leveraging existing inventories to produce metrics that best approximate sources of point and non-point source pollution to surface water through a simplified framework can allow decision-makers and stakeholders alike to develop efficient and effectual watershed restoration plans.

The most important aspect of prioritization using inventories is to identify areas where large amounts of surplus anthropogenic nitrogen and phosphorus are left in the landscape. This metric, variously derived, largely determines the magnitude of nutrient loss to streams and rivers across time and space (Carpenter *et al* 1998, Howarth *et al* 2012, Hong *et al* 2013, Chen *et al* 2016). However, recent inventories also allow insight into whether urban or agricultural interventions decrease or at least attenuate surplus nutrients across the landscape (Sabo *et al* 2019). The most effective interventions will likely revolve around three basic actions: increase nutrient use efficiency in agricultural production in order to decrease nutrient surplus in agricultural fields (e.g., nutrient management plans, manure transport programs (Davidson *et al* 2016)), (2), decrease nutrient inputs (e.g., lawn fertilizer, atmospheric deposition) (Eshleman and Sabo 2016, Hobbie *et al* 2017), and (3) upgrade wastewater treatment facilities to remove nutrients from human associated household and industrial waste effluent (Chanat and Yang 2018, Stets *et al* 2020). Within a watershed, the combination of aforementioned actions and societal investments can be enhanced through proposed trading mechanisms, progress can be tracked with easily understood indicators based on existing inventories (McLellan *et al* 2018).

Leveraging recently compiled nitrogen and phosphorus inventories for the years 2002, 2007, and 2012 (Sabo *et al* 2019, Sabo *et al* 2021) with a point source loading database (Ivahnenko 2017), we extracted relevant fluxes (e.g., farm fertilizer, human food demand, etc) to help identify likely US subbasins (Hydrologic Unit Codes-8, ≈1,800 km^2^) contributing a disproportionate amount of point and non-point source nutrient loads to surface waters by exploring the mean spatial distribution of anthropogenic N and P surplus, agricultural N and P surplus, and point source N and P loads. Intensive agricultural production as well as the distribution of industrial and municipal wastewater treatment plants are known to be concentrated in specific regions of the country (Sabo *et al* 2019), thus, we expect that only a small portion of the landscape across the United States holds the majority of point source loads and agricultural surpluses likely responsible for nutrient pollution to surface waters. Identification of these areas along with relevant data on the magnitude of fluxes and surpluses provide a convenient platform for decision makers to identify the predominant source of nutrients likely driving water quality degradation and highlight potential avenues to most efficiently and effectively achieve water quality goals.

Here we use the N and P inventories to overlay sub-basin areas of low nutrient use efficiency with areas of high values of nutrient surplus across the contiguous United States (CONUS). The inventory data also allows us to visualize where point sources are the dominant source. By combining these key inventory metrics, we can identify priority sub-basins within the CONUS that have high nutrient loads combined with the opportunity for improvement in nutrient management using current reduction tools.

## Methods

### Input data from existing inventories

In order to derive anthropogenic N and P surplus, agricultural N and P surplus, and point source N and P loads, relevant fluxes were extracted from recently compiled nutrient inventories for the 2002–2012 period (Sabo *et al* 2019, Sabo *et al* 2021). Human N and P demand, non-farm N and P fertilizer, agricultural N and P fertilizer, cultivated biological N fixation, crop N and P removal, livestock N and P feed demand, livestock N and P production, and atmospheric NO_x_ deposition values were extracted from recent USGS Hydrologic Unit Code subbasin scale nitrogen and phosphorus inventories for the years 2002, 2007, and 2012 (Sabo *et al* 2019, Sabo *et al* 2021). Here, we briefly summarize the methods for the N and P inventories and refer the reader to Sabo *et al* 2019 and 2021 for more detailed information (table S1 (available online at stacks.iop.org/ERC/3/045005/mmedia)). Human N and P demand were based on US Census block population data and specific food and non-food demand constants (Sabo *et al* 2019, Sabo *et al* 2021). Livestock production and feed demand/waste/production relied on Census of Agriculture (CoA) county level livestock and poultry population data and were based on a commonly applied static livestock model (Boyer *et al* 2002, Sabo *et al* 2019). Crop N and P removal estimates, which is the mass of N and P removed from fields following harvest, was based on CoA crop yield data and crop specific removal coefficients as was cultivated biological N fixation (Fixen *et al* 2012). Total atmospheric NO_x_ deposition estimates were based on a hybrid of modeled and observed wet and dry atmospheric deposition rates (Schwede and Lear 2014).

Farm and non-farm fertilizer rates were extracted from a periodically updated USGS database and are based on a combination of state/county level fertilizer sales data, county level chemical farm expenditure data from CoA, and effective human population size (Gronberg and Spahr 2012, Brakebill and Gronberg 2017). It should be noted that Sabo *et al* 2019 relied on farm N fertilizer estimates from Fixen *et al* (2012) in the N inventory whereas the P inventory relied on Brakebill and Gronberg 2017, thus to allow for consistency, farm N and P fertilizer rates from the USGS report were used in this analysis (Brakebill and Gronberg 2017). In addition, HUC-8 N inventory values were transferred to the most recent version of the HUC-8 spatial data layer as used in Sabo *et al* 2021 to ensure further comparability among the nutrient inventories for this analysis. Four subbasins from the N inventory were dropped in the newest iterations (the water bodies of the Great Lakes) and large water bodies and international areas within the HUC-8 were no longer calculated as the area of the subbasin. Flux estimates were maintained by transferring the areal normalized values to the updated HUC-8 file, largely maintaining a strongly correlated, 1:1 correspondence between new and old inventory values. Industrial and municipal point source N and P loads for 2004, 2008, and 2012 were extracted from a recently released United States Geologic Service database by intersecting wastewater treatment plant locations within the subbasins with GIS software (Ivahnenko 2017). The inventory and point source fluxes described above were averaged to capture the mean conditions from 2002–2012 period.

### Derived variables and ranking

Separately, we calculated the anthropogenic N and P surpluses for all subbasins across the country. This metric simply reflects the difference between inputs/demand (human N and P demand, non-farm N and P fertilizer, agricultural N and P fertilizer, cultivated biological N fixation, livestock N and P feed demand, atmospheric NO_x_ deposition) and outputs (livestock N and P production, crop N and P removal). Anthropogenic N and P surplus were inspired by net anthropogenic nitrogen and phosphorus inputs, which have been shown to be effective predictors of the spatiotemporal variability of surface water nutrient export (Han *et al* 2011, Hong *et al* 2012, Howarth *et al* 2012, Hong *et al* 2017). The anthropogenic surplus, however, does not apply the handling loss constants applied after crop removal and livestock production used to finalize the calculation of the net anthropogenic nitrogen and phosphorus inputs (oftentimes ∼10%) (Hong *et al* 2011).

While anthropogenic surplus captures net inputs or surplus within a subbasin, the agricultural N and P surplus captures balances in the integrated soil-plant system of groups of farms within a subbasin (Zhang *et al* 2015, Zhang *et al* 2020). The agricultural N surplus is simply the difference between farm associated inputs (fertilizer, atmospheric NO_x_ deposition onto farmland, livestock waste, cultivated biological N fixation) and crop removal. The calculation is similar for agricultural P surplus except there are no fixation or deposition input terms. As one would expect (Hong *et al* 2013, Hong *et al* 2017), the anthropogenic surplus and agricultural surplus are highly correlated across the CONUS (r^2^ = 0.76 and 0.65 for N and P, respectively, Supplemental Database), and even more so in highly agricultural areas like subbasins in Iowa (r^2^ = 0.96).

In addition, the ratio of agricultural outputs and inputs was calculated to determine the nutrient use efficiency for N and P (NUE) in all subbasins. The agricultural surplus and NUE for N and P are more meaningful metrics for farmers and decision makers because they highlight areas of inefficient nutrient use and likely significant annual nutrient accumulation in agricultural settings across the CONUS. We also derived the efficiency in decreasing agricultural surplus by a 1% gain in NUE. This was accomplished by applying a hypothetical scenario where all subbasins with NUE <90% were raised to 90%; from there, the absolute decline in surplus was divided by the absolute change in NUE. Overall, this derived efficiency highlights subbasins where agricultural inputs are largest thus greater declines in surplus can be achieved with small increases in NUE. This metric reveals which catchments would be most efficient in decreasing surplus per unit gain in NUE (Quemada *et al* 2020). Likewise, we focused our efforts on illustrating the intensity of point source loads across the CONUS to identify areas of country where further enhancements to wastewater treatment technologies could be beneficial to local water ways.

Before ranking all individual fluxes and derived metrics, all mass flux and surplus values (kg yr^−1^) were normalized by the subbasin area. This normalization serves two purposes. First, strong and generally consistent relationships between areal normalized inputs/surpluses and stream nutrient export have been suggested throughout the literature (Howarth *et al* 2012, Chen *et al* 2016, Chen *et al* 2018). While users without water quality observations in their local area can approximate water quality responses to shifts in surpluses or inputs based on these relationships, it would be optimal to determine watershed level relationships between the mass balances in this database (or the parent nitrogen and phosphorus inventories, (Sabo *et al* 2021, Sabo *et al* 2021)) as the variation in retention can be quite variable (Howarth *et al* 2012). For example, recent observations and modeling work in Germany found 95% of the N surplus may be denitrified along groundwater pathways before even reaching surface water, while other catchments showed little to any capacity to remove N surplus through subsurface pathways. This database can thus be used for management and further research purposes to explore relationships between surplus and stream export and identify factors that modify this relationship (Knoll *et al* 2020). Second, ranking subbasins only by mass of N and P would likely lead to larger basins being ranked higher than smaller basins largely due to the difference in area rather than the intensity of fluxes or surpluses. Both the mass of nutrients and areal normalized values can be generated in the supplemental database depending on user preference, but for this report we focused on areal normalized rankings to better highlight likely pollution hot spots across the CONUS. This database includes state tags so that state level decision makers can quickly filter to explore the magnitude of fluxes and surpluses for subbasins that occur within their jurisdiction. As such, the rankings and magnitude of fluxes and surpluses can be explored from the national, state, and multiple watershed scales. Ultimately, this database allows the user to customize their rankings in order to explore their (1) scale of interest (down to the subbasin scale) and (2) pollution source of interest.

## Results and discussion

### Broad patterns of likely areas of point and non-point source nutrients

**Figure 1.**
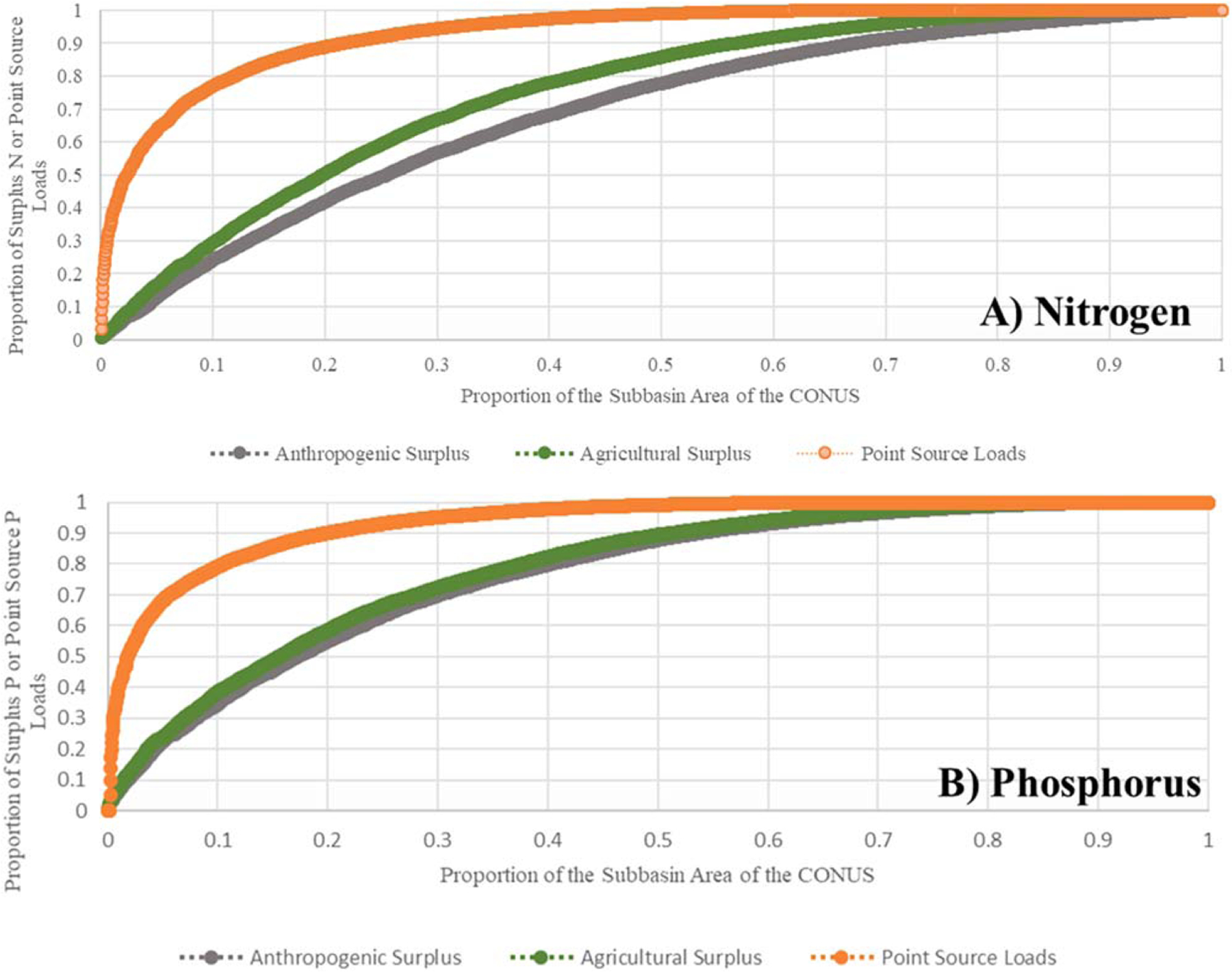
Proportion of the land area of the CONUS holding the amount of anthropogenic surplus and agricultural surplus nutrients as well point source loads.

A small proportion of the landscape is responsible for the majority of point and non-point source N and P pollution to surface waters across the contiguous United States. Indeed, 50% of the sources of N pollution are associated with 2%, 20%, and 25% of the subbasin area for point sources, agricultural surpluses, and anthropogenic surpluses, respectively (figure 1(A)). For P, 2%, 17%, and 18% of the subbasin area contains 50% of pollution sources associated point sources, agricultural surpluses, and anthropogenic surpluses, respectively (figure 1(B)). The fraction of the CONUS landscape likely contributing to point and non-point source pollution is actually even smaller considering the fact that agricultural and urban pollution sources are associated with only specific land uses within these subbasins (e.g., agricultural surplus only occurs on agricultural land). These findings highlight that concerted efforts to constrain N and P pollution to surface water may be more effectively achieved by targeting only a small proportion of a watershed for restoration (McCrackin *et al* 2018, Spiegal *et al* 2020, Zhang *et al* 2020), thus allowing more efficient use of resources and effort to identify areas for restoration to achieve downstream water quality goals. These findings are consistent with both recent nation-wide and regional water quality analyses highlighting nutrient pollution hot spots across the landscape (Ator 2019, Hoos and Roland II 2019, Robertson and Saad 2019, Wise *et al* 2019), but use of available inventory data highlights the magnitude of likely drivers of water quality degradation and offers a convenient platform with management relevant metrics for decision makers to (1) identify candidate subbasins for restoration and (2) work with local stakeholders to craft restoration goals (Sobota *et al* 2013, Sabo *et al* 2021).

**Figure 2.**
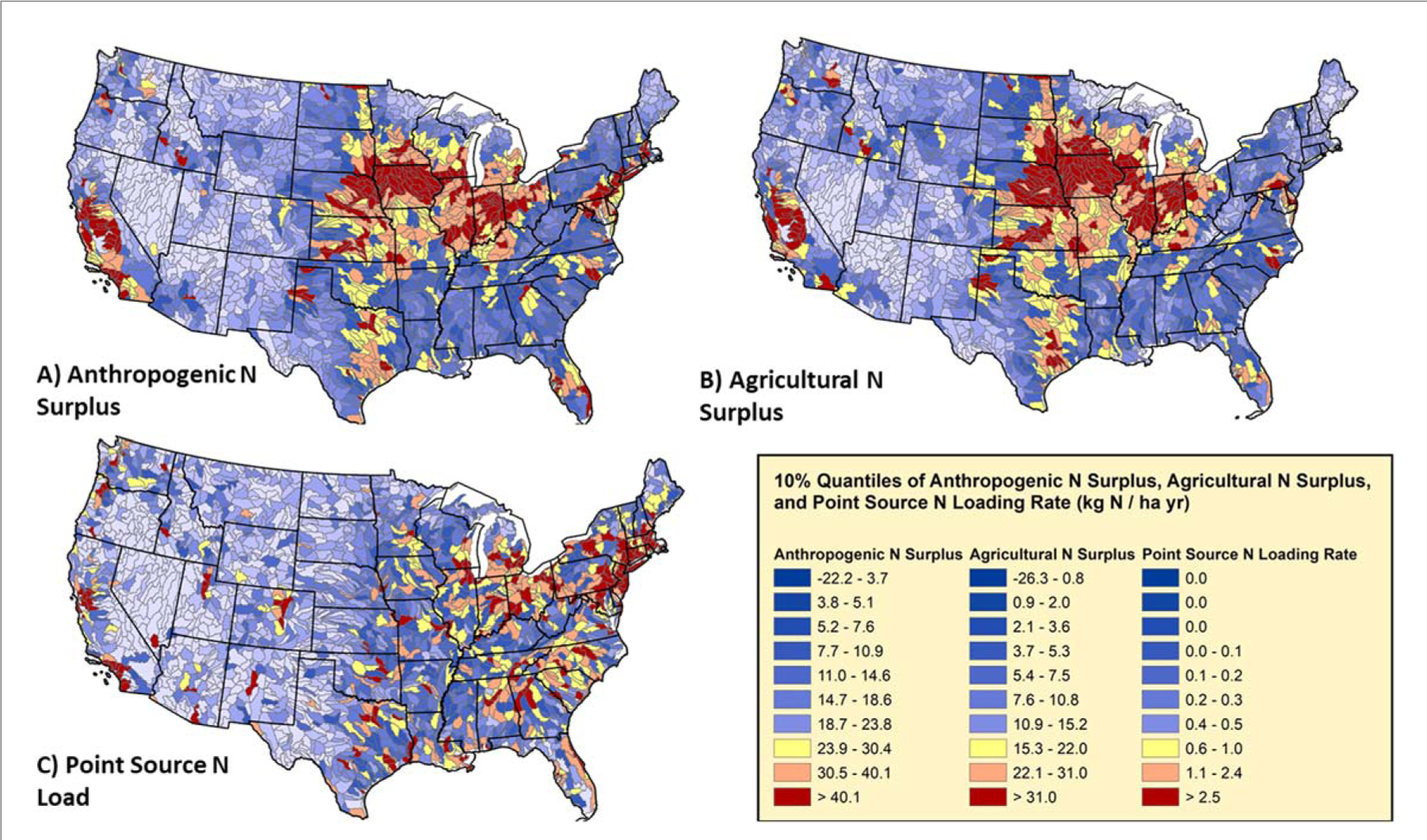
Average anthropogenic and agricultural N surpluses as well as point source loads for all subbasins across the CONUS from ∼2000–2012 ordered by 10% quantiles. Areas in red have the highest surpluses and point source loads, light blue the lowest. Please note, that 53 subbasins with the highest point source loads, in and around major population centers, have rates in excess of 10 kg N ha^−1^ yr^−1^.

**Figure 3.**
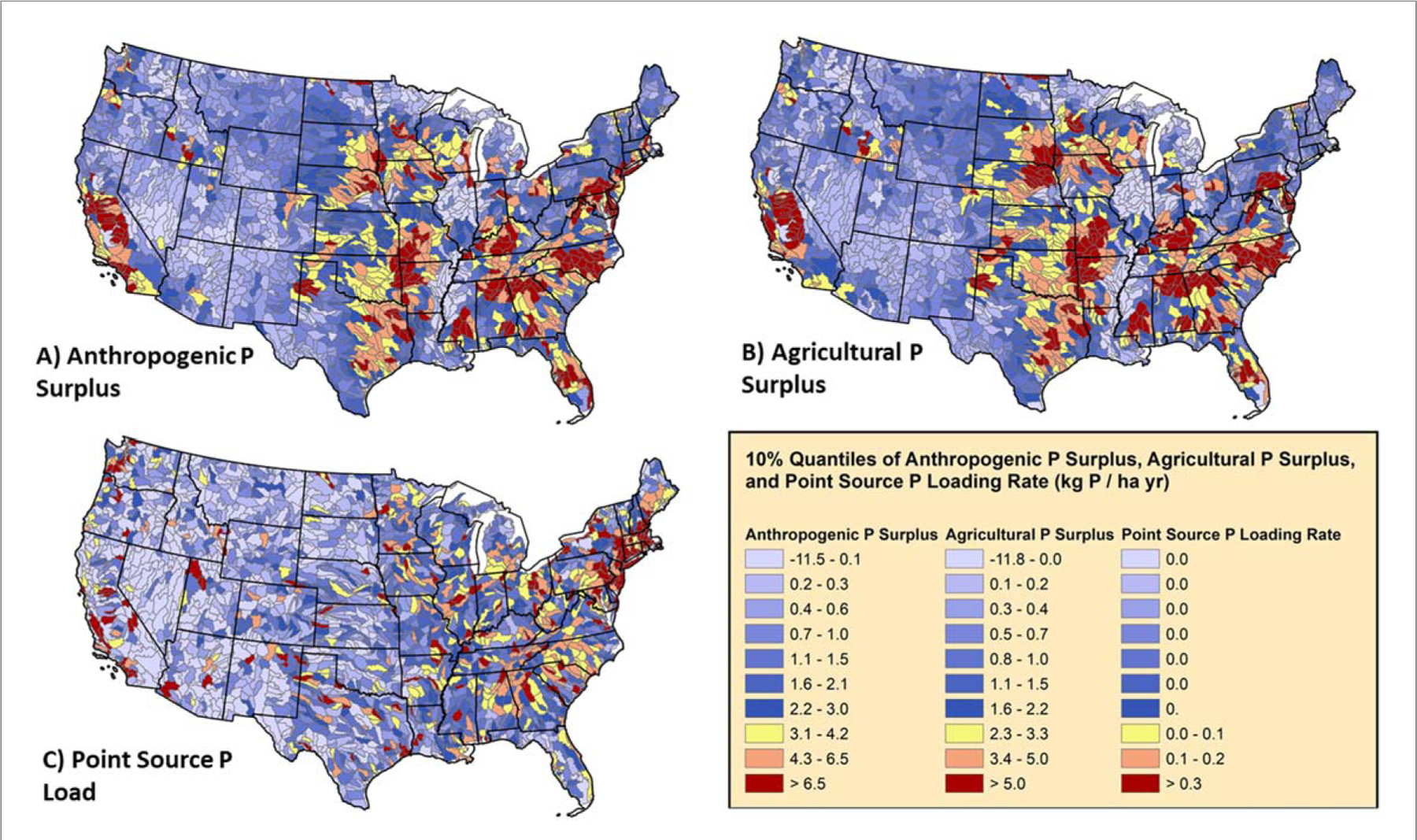
Average anthropogenic and agricultural P surpluses as well as point source loads for all subbasins across the CONUS from ∼2000–2012. Areas in red have the highest surpluses and point source loads, blue the lowest. Please note, that 41 subbasins with the highest point source loads, in and around major population centers, have rates in excess of 1.0 kg P ha^−1^ yr^−1^.

Anthropogenic surpluses for both N and P generally parallel spatial patterns of agricultural surpluses (table 1, r = 0.71 and 0.81, respectively) highlighting the likely disproportionate influence that agriculture has on nutrient pollution on surface waters throughout most of the country (figures 2(A)–(B), 3(A)–(B)). Both anthropogenic and agricultural surpluses are highest in some of the most productive agricultural regions of country—the Upper Midwest, Great Valley of California, southeastern North Carolina, and parts of the Chesapeake Bay watershed, as observed in previous studies (Swaney *et al* 2018, Swaney and Howarth 2019). However, many other subbasins generally surrounding these hotspots have agricultural surpluses >15 kg N ha^−1^ and >2 kgP ha^−1^ thus efforts to decrease agricultural surpluses in these basins have the potential for decreasing anthropogenic nutrient surplus and nutrient loads to surface water as well (figures 2(B), 3(B)). Surprisingly, agricultural N and P surpluses are generally correlated (r = 0.51, table 1), but not as strongly correlated as one would expect considering the importance of these macronutrients in maintaining crop yields. This suggests state-specific efforts to improve nutrient management as well as regionally specific trends in agricultural are resulting in variable impacts on agricultural surpluses. One of the intriguing deviations between agricultural N and P surpluses, is the consistently low or even negative P surpluses centered on Illinois and Iowa versus comparatively high agricultural N surpluses (figures 2(B) and 3(B)). These deviations may reflect recent state efforts to improve nutrient use efficiency, which seem to be effective for decreasing phosphorus surpluses on agricultural land (Swaney *et al* 2018, Swaney and Howarth 2019).

Recent regional application of spatially-referenced regression models across the CONUS have also inferred the disproportionate influence agricultural production has on downstream water quality with agricultural land use, fertilizer, cultivated biological N fixation, and/or manure excretion consistently being the top and some of most influential explanatory variables of anthropogenic nutrient loads for all regional models (Sinha and Michalak 2016, Ator 2019, Robertson and Saad 2019). Inference from the latter studies and this one is consistent with past studies (Galloway *et al* 2003, Galloway *et al* 2008, Doering *et al* 2011), yet this inventory makes an actionable metric available, agricultural surplus, that farmers within a subbasin can improve with best nutrient management practices and continued innovation in fertilizer technologies and crop cultivars (Zhang *et al* 2015, Davidson *et al* 2016, Houlton *et al* 2019). Respectively, about 67% and 80% of the anthropogenic N and P surplus is attributable to the N and P left in fields and pastures across the country with the remainder being attributed to NOx deposition on non-agricultural land, lawn fertilizer application, and human food and non-food demand. Similar proportions have been observed in other national inventories or inferred from SPARROW models (Doering *et al* 2011, Houlton *et al* 2018, Robertson and Saad 2019). While nutrient use efficiency has greatly increased over the past four decades (Zhang *et al* 2015, Lu *et al* 2019), farmer led efforts to further increase nutrient use efficiency and decrease nutrient surpluses on cropland and pasture will be instrumental in achieving both local and nationwide water quality goals.

**Figure 4.**
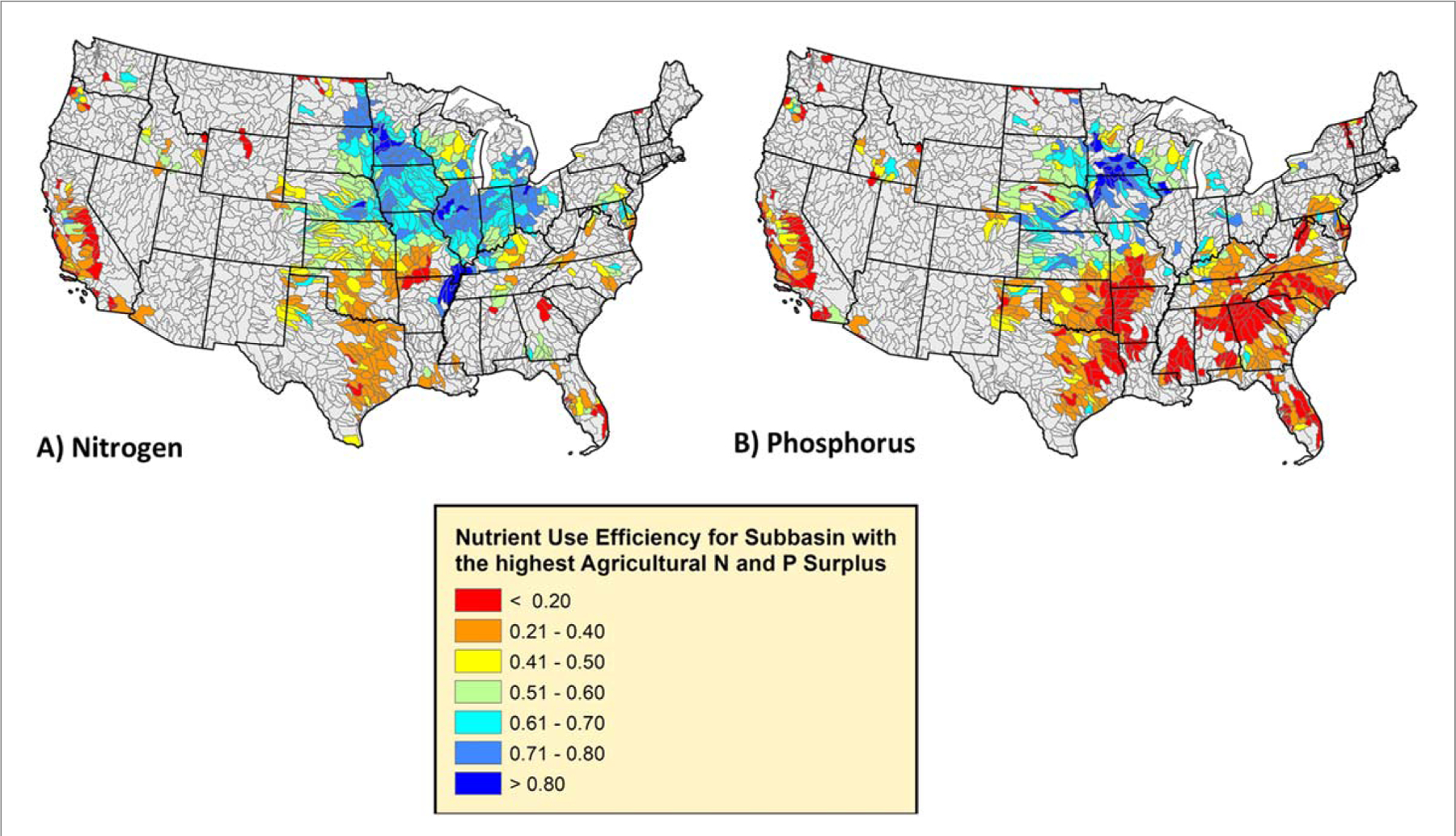
Agricultural nutrient use efficiency in subbasins with the highest agricultural N and P surplus (70th–100th percentiles, the yellow, orange, and red areas, from figures 2(b) and 3(b)).

The magnitude of agricultural surpluses is an important consideration when prioritizing catchments for restoration, in addition to mean nutrient use efficiency (Schröder *et al* 2011, Fixen *et al* 2012, Smith *et al* 2017). While efforts to attain unity in input and crop removal rates within a field or catchment is ideal, the effort, as well as the risk to farmers to reduce surplus yet not decrease yield, increases as farmers lower fertilizer inputs (Zhang *et al* 2015, Davidson *et al* 2016, Zhang *et al* 2020). Thus, identifying areas of the country, state, or watershed where nutrient use efficiency could be feasibly and efficiently increased with adjustments in fertilizer and manure use to meet local crop needs is needed (figures 4(A)–(B), S1) (Schröder *et al* 2011). For a simple illustration, the majority of the subbasins in Iowa are in the top 10% for agricultural N surplus as are large portions of Kansas and Nebraska (red areas, figure 2(B)). However, the Iowa subbasins have mean nutrient use efficiency values consistently 10%–30% higher than regions further west. Certainly, there are climatological limitations to yields in these more western, arid subbasins (Lu *et al* 2019), but significant improvements in NUE and surplus may be achieved by scaling back fertilizer and manure inputs without compromising crop yield, since inputs largely exceed crop yields. Especially for P, many subbasins in the South and Southeast have extremely low NUE due to extensive livestock production with relatively low crop removal rates (figures 4, S1). This observation highlights the more complex task of optimizing livestock-crop producing systems where currently a large proportion of grain is imported to feed poultry and livestock (Spiegal *et al* 2020).

**Figure 5.**
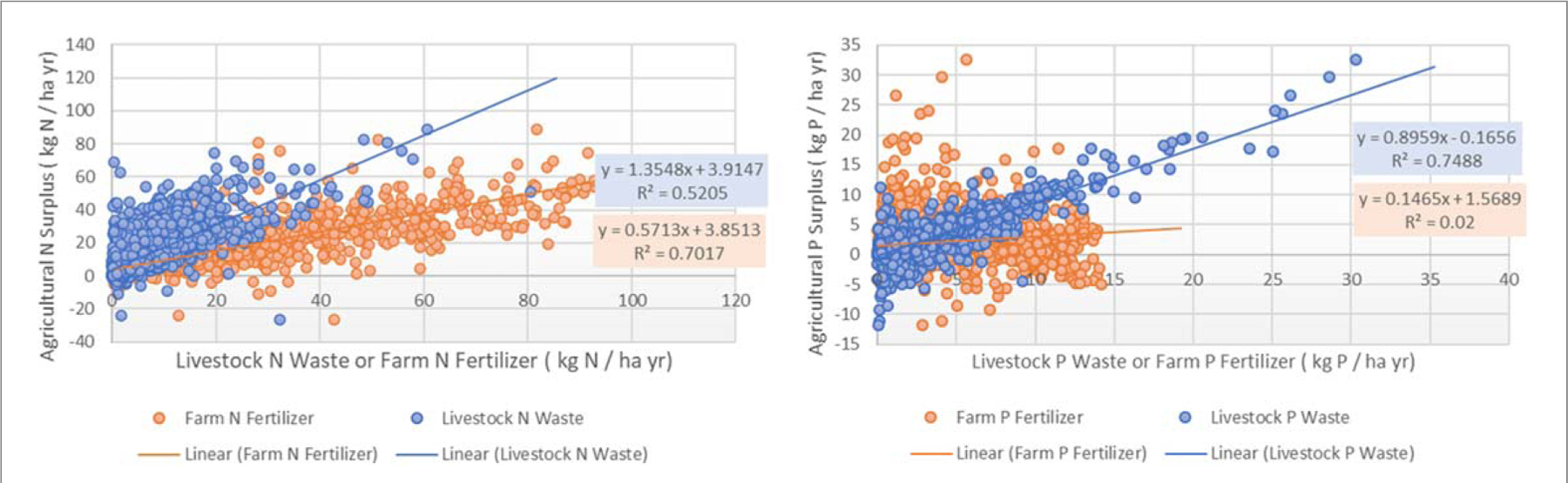
Relationship between farm N and P fertilizer and livestock N and P waste rates versus annual agricultural N and P surplus. Each data point on the scatter plot represents an individual subbasin.

Generally, only a few locations in the United States are even close to experiencing nutrient mining under current cropland management strategies (Fixen *et al* 2012), thus farmers residing within subbasins throughout the Midwest and other subcatchments currently in the top 30% for largest agricultural surpluses (non-blue areas; figures 2(B) and 3(B)) could substantially improve downstream water quality by increasing nutrient use efficiency (figure 4). Primarily agricultural catchments within the Chesapeake Bay that have increased NUE and decreased N and P surpluses have seen parallel relative declines in nutrient export (Devereux and Rigelman 2014, Moyer 2016, Sabo 2018, Fanelli *et al* 2019). Furthermore, other watersheds have been generally responsive to changes in surplus nutrients across time throughout Europe and the United States (Sinha and Michalak 2016, Hong *et al* 2017). By and large, surplus nutrients can be attenuated with better use of manure nutrients, as this source of nutrients has been considered to be treated as a waste issue rather than as a nutrient resource (Swaney *et al* 2018, Swaney and Howarth 2019, Spiegal *et al* 2020). This is most clearly illustrated for P, where fertilizer has little if any relationship with the agricultural P surplus, but livestock waste has a strong linear relationship (figures 5(B), S1). However, a positive insight into these relationships highlights that primarily crop producing regions of the country that rely on farm P fertilizer are amending soils to meet crop needs and these areas (e.g., much of Illinois and Iowa) have indeed observed declines in surface water TP loads and concentrations from 2002–2012 (Oelsner *et al* 2017). For the rest of the country, if the magnitude of nutrient inputs from manure were reduced by 50% (or more realistically a corresponding reduction in fertilizer with reallocated livestock waste), agricultural surpluses across the CONUS could decrease from 9 Tg to 7 Tg for N and 1.46 Tg to 0.56 Tg for P. Efficiently allocating manure nutrients to meet crop nutrient demand with corresponding declines in fertilizer use could make significant in-roads for achieving nation-wide water quality goals (Spiegal *et al* 2020). While this analysis does not prescribe specific solutions for locales, farm nutrient management plans may be an effective component (Schröder *et al* 2011, Davidson *et al* 2016). The database can be used by decision makers to help identify portions of landscape where nutrient management strategies, developed with local stakeholders, are likely to meet local nutrient management goals.

As others have observed (Doering *et al* 2011, Houlton *et al* 2013, Houlton *et al* 2019), agricultural lands hold the majority of anthropogenic surplus N and P across the landscape, but urban domains can also have outsized influence on surface water nutrient loads (Gao *et al* 2014, Hong *et al* 2017, Stets *et al* 2020). Recent nationwide trends research suggests that more urbanized catchments across the United States have shown declines in N and P export (Stets *et al* 2020), whereas trends in agricultural catchments and undisturbed catchments have been more variable (Oelsner *et al* 2017). These improvements in water quality in urban areas are likely tied to success in decreasing atmospheric N deposition by reducing NO_x_ emissions from power plants and vehicles (Eshleman *et al* 2013, Eshleman and Sabo 2016) and efforts to upgrade wastewater treatment plants with advanced nutrient removal technologies (Hirsch *et al* 2010, Ator *et al* 2019). While declines in atmospheric NO_x_ deposition directly decrease anthropogenic N surplus in the terrestrial compartment in turn likely decreasing non-point source loads (Hobbie *et al* 2017), point source loads actually represent a downstream flow of anthropogenic N and P (via pipes) that can be mitigated by wastewater treatment plants (Diaz 2001). Since standardized estimates of point source loads are becoming more available across space and time (USEPA 2015, Ivahnenko 2017), ranking watersheds by point source loads rather than solely by human food and non-food demand may actually provide more relevant information as these estimates directly incorporate the impact of facility specific technologies attenuating human and industrial waste (Shenk and Linker 2013).

Point source loads are primarily concentrated in major metropolitan regions across the country with point source loading rates exceeding >10.0 kg N ha^−1^ yr^−1^ and >1.0 kg P ha^−1^ yr^−1^ in many subbasins (a subset of red areas, figures 2(C) and 3(C)). The areal normalized rates for these subbasins are high and targeted upgrades to further decrease effluent concentrations could have an outsized impact on improving local water quality and maybe even in larger mixed land use basins like the Chesapeake Bay (e.g., (Ator *et al* 2019)). Improvements in waste treatment technologies can be beneficial outside of major cities, since the presence of industrial plants (e.g., paper mills, breweries, chemical factories) or municipal wastewater treatment plants with only primary treatment technologies may be a large source of nutrient pollution to local waterways (Shenk and Linker 2013). However, the decision to allocate resources to upgrade a given wastewater treatment should be weighed against the influence of non-point source loads in a given catchment (Sabo *et al* 2021). To be clear, point source loads currently only account for a small proportion of the anthropogenic N and P surplus across the United States with the remainder tied to non-point source loads from urban, agricultural, and natural catchments. While wastewater treatment plant upgrades can offer near immediate improvements in water quality (Hirsch *et al* 2010), oftentimes even the maximum theoretical reduction in point source loads in a catchment, especially larger catchments, are nowhere near the reductions needed to achieve water quality restoration goals (Sabo 2018, Robertson and Saad 2019).

### An example of national watershed rankings

Prioritizing watersheds for restoration are dependent on a myriad of factors including the scale of wider watershed restoration objectives, jurisdictional authority of decision makers, available resources and tools, as well as the magnitude of point and non-point source pollution sources within a local area. This study does not prescribe an absolute ranking that is applicable for the nation because local priorities and factors may drive different criteria for different areas. However, this study provides a simple national example on how to identify subbasins for potential restoration. The methodology allows for a large number of ‘customizations’ that can be applied at the national, state, and watershed scale. The database associated with this effort (Supplemental Database) is structured in a way that a decision-maker can filter and organize ranks of watersheds according to local conditions using standard and widely available software.

Prioritizing subwatersheds for restoration will highlight likely areas of the country, state, or watershed where reductions in point and non-point source pollution can be most efficiently and effectively achieved. Agriculture may hold significant promise in delivering further reductions in nutrient loads. However, dependent on the scale of restoration, many urbanized subbasins can still achieve significant nutrient reductions via further upgrades to more advanced wastewater treatment technologies or sewage infrastructure improvements (e.g. elimination of combined sewage overflows). From a national perspective, any watershed falling in the greater than >90th percentiles in point-source surpluses can lead to significant improvements in nutrient loads to local surface waters (red areas, figures 2(C) and 3(C)) with wastewater treatment upgrades (Hirsch *et al* 2010, Fanelli *et al* 2019). Before pursuing these infrastructure improvements, two major considerations should be considered. First, what are the existing treatment levels for wastewater treatment plants within the subbasin and can they be feasibly upgraded by the local jurisdiction? While existing treatment levels are either directly reported or can be inferred using the Hypoxia Task Force point source loading tool (USEPA 2015), cost estimates for upgrades are well beyond the scope of this work and are not readily available. The second and potentially even more important piece of information to consider, is whether or not reductions in nutrient loads to surface waters could be more efficiently achieved via reductions in non-point source loads from urban fertilizer, atmospheric deposition, and agricultural surpluses (Klages *et al* 2020, Stoner 2011). Indeed some of the biggest improvements in nutrient loads have resulted from efforts to decrease nutrients loads from agriculture (Dalgaard *et al* 2014, Chanat and Yang 2018, Ator *et al* 2019).

**Figure 6.**
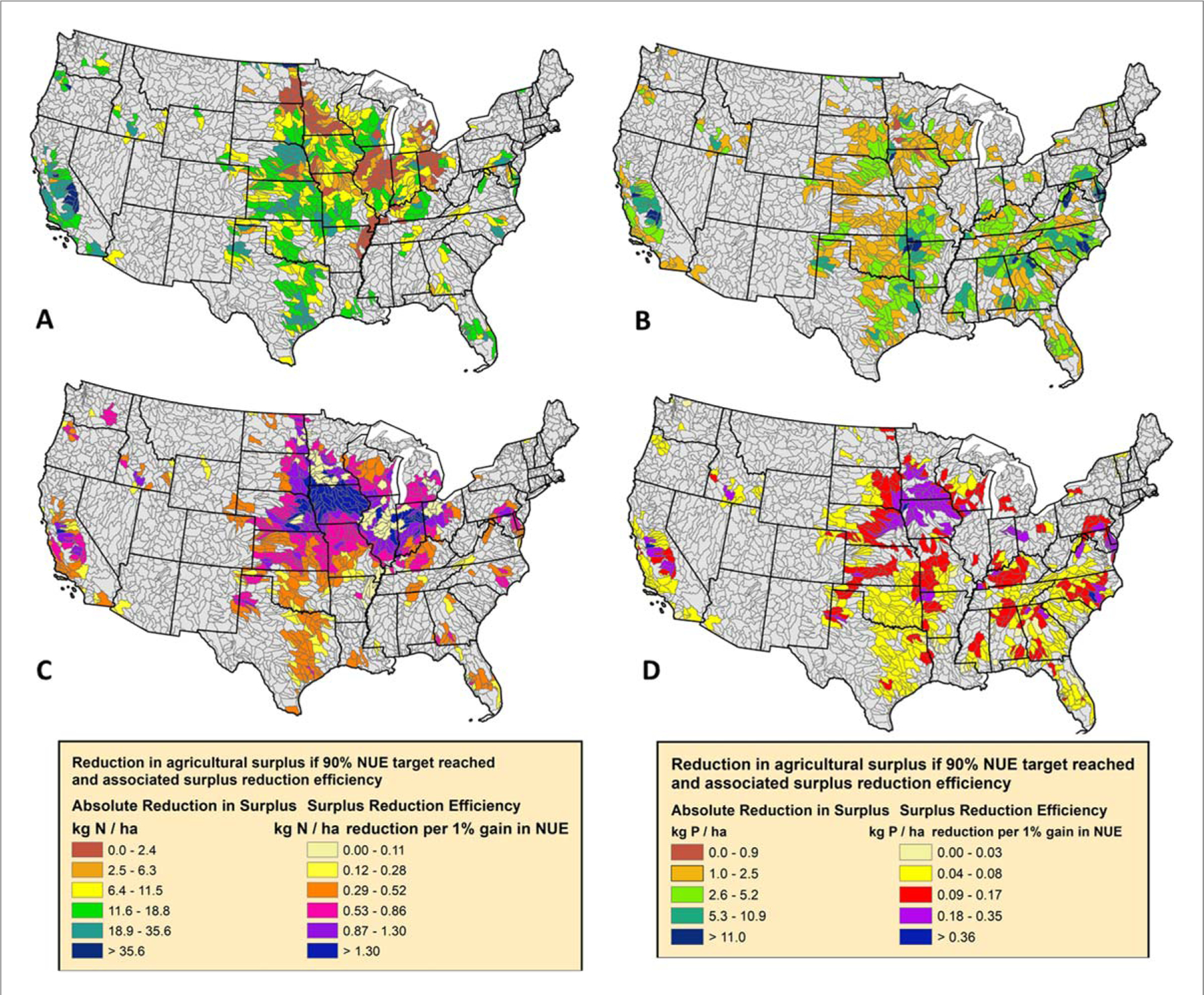
Absolute reductions in agricultural nitrogen and phosphorus surplus if nutrient use efficiency was raised to 90% for all subbasins across the CONUS and surplus reduction efficiency. For illustrative purposes, only subbasins with the highest agricultural surpluses, in the 70th to 100th percentiles, are shown (yellow, orange, and red areas in figures 2(B) and 3(B)).

From a national perspective, subbasins falling within the 70–100th percentiles of agricultural surplus have a disproportionately degrading influence on downstream water quality (non-blue areas, figures 2(B) and 3(B)). Surprisingly, some of these subbasins already have NUE values exceeding 80%, thus the feasibility to decrease agricultural surpluses further without the risk of nutrient mining and compromising crop yield and quality declines (∼90% NUE, (Dalgaard *et al* 2014, Fixen *et al* 2012, Quemada *et al* 2020)). Thus, we recommend not only considering agricultural surpluses and NUE, but also the potential reduction in agricultural surplus if hypothetical NUE is optimized to a target. For illustrative purposes, we chose 90% efficiency, but this target value depends highly on local, achievable agricultural practices (figures 6(A)–(B)) (Fixen *et al* 2012, Dalgaard *et al* 2014, Quemada *et al* 2020). In addition to surplus improvement, the efficiency in reducing the surplus per 1% gain in NUE (figures 6(C)–(D), simply the absolute change in surplus divided by the increase in NUE to reach 90%) should be considered. After these additional nuances, it is clear that a large fraction of agricultural N surpluses in this cohort would not be greatly reduced with increases in NUE (brown and dark yellow areas, figure 6(A)) since efficiencies were already close to or exceeding 90% (Illinois, souther Minnesota, figure 6(B)). NUE should continue to be optimized in these subbasins but other management actions to mitigate non-point source loads and potentially legacy nutrients in the soil may need to be considered (Chen *et al* 2018). Other much larger declines in agricultural N and P surpluses could be achieved but the efficiency is variable due to local practices tied to crop production, manure generation, and fertilizer use (figures 6(C)–(D)). Productive crop regions like Iowa show the highest efficiencies in decreasing surplus (purple and blue areas, figures 6(C)–(D)), but declines in agricultural nutrient surplus there are oftentimes < 50% compared to predominantly livestock producing regions with less efficiency gains (e.g., Chesapeake Bay, southeastern North Carolina).

Nutrient management plans and outreach to farmers have been shown to be a cost-effective means to increase NUE and even decrease surpluses in some locales over the last four decades (Devereux and Rigelman 2014, Davidson *et al* 2016). However, some agricultural regions focused on livestock and poultry productions are saddled with manure and poultry litter nutrients that cannot be effectively utilized by current crop removal rates since the majority of N and P is imported from outside the region (Spiegal *et al* 2020). As some have highlighted, this valuable nutrient resource is being treated more as a waste product (Swaney *et al* 2018, Swaney and Howarth 2019). Water quality improvements can be substantial if surpluses can be attenuated in these more manure-driven subbasins (figure S1), but the efficiency to decrease those surpluses may be cost prohibitive to individual farmers. Some states have attempted to implement water quality trading schemes to help either transport manure out of these watersheds or generate value added products to increase the efficiency of manure use and decrease surplus (Stoner 2011, Devereux and Rigelman 2014, Stephenson and Shabman 2017), but success has been limited due to constricted markets and difficulty in attributing credits (Dotterer 2017, Stephenson and Shabman 2017, Klages *et al* 2020). This standardized inventory could help facilitate broader markets and provide standardized metrics to trade potential point and non-point source credits (Dotterer 2017, Stephenson and Shabman 2017, Klages *et al* 2020).

## Conclusions

This analysis highlights that a small proportion of the landscape contains significant quantities of anthropogenic surplus nitrogen and phosphorus. These surpluses are often co-located, especially in areas of extensive livestock production. Most of the surplus N and P degrading water quality is from unutilized fertilizer and manure nutrients left on cropland and pasture. Farmers in many regions of the country have increased nutrient use efficiency and decreased agricultural surplus via (1) simple adjustments to farm level nutrient management and (2) unanticipated reductions in atmospheric NO_x_ deposition (Zhang *et al* 2015, Lu *et al* 2019). Further progress in boosting nutrient use efficiency in these areas as well as other areas where efficiency is low, as identified in this analysis, could contribute to large gains in water quality restoration goals for relatively low costs (Devereux and Rigelman 2014, Stephenson and Shabman 2017). Concerns about legacy nutrients in the soil and groundwater delaying water quality improvement are important and factor into expectations for watershed recovery (Van Meter *et al* 2017, Chen *et al* 2018). Point source loads are especially relevant sources of nutrient pollution in certain locales, and further wastewater treatment plant upgrades can be useful for achieving restoration goals and mitigating future pollution due to increased population in certain subbasins (Sabo *et al* 2021, Sabo *et al* 2021). The ability of this analysis and the corresponding database to integrate multiple information sources to provide estimates of fluxes and surpluses across urban and agricultural domains as well as highlight areas where N and P are being managed inefficiently gives decision makers a succinct platform to identify likely areas of water quality degradation. This quantitative information can be integrated into prioritization efforts to restore watersheds and improve water quality.

## Supplementary Material

S2

S1

## Figures and Tables

**Table 1.** Pearson correlation among primary ranking metrics to evaluate likely point and non-point source contributions to surface water.

	Anthropogenic N Surplus	Point Source N Loading	Agriculture N Surplus	Nutrient Use Efficiency, N	Anthropogenic P Surplus	Point Source P Loading	Agriculture P Surplus	Nutrient Use Efficiency, P

Anthropogenic *N* Surplus	1							
Point Source N Loading	0.52	1						
Agriculture N Surplus	0.70	−0.01	1					
Nutrient Use Efficiency, N	−0.03	−0.02	<0.01	1				
Anthropogenic P Surplus	0.73	0.43	0.43	−0.08	1			
Point Source P Loading	0.04	0.09	−0.02	−0.01	0.036	1		
Agriculture P Surplus	0.39	−0.02	0.51	−0.08	0.81	−0.01	1	
Nutrient Use Efficiency, P	0.02	−0.03	0.11	0.23	−0.35	−0.01	−0.41	1

## Data Availability

All data that support the findings of this study are included within the article (and any supplementary files).
