# Supplementary material for: Considerations when using nutrient inventories to prioritize water quality improvement efforts across the US
Source: Environ Res Commun. Author manuscript; Available in PMC 2022 Nov 30. (PMC9709726; doi:10.1088/2515-7620/abf296)
Supplement: S2 [file NIHMS1701162-supplement-S2.pdf]

Title

Considerations when using nutrient inventories to prioritize water quality improvement efforts across the US

Authors

Robert D. Sabo[1], Christopher M. Clark[1], Jana E. Compton[2]

[1] Center for Public Health and Environmental Assessment, Office of Research and Development, U.S. Environmental Protection Agency, Washington, D.C., USA; [2]Center for Public Health and Environmental Assessment, Office of Research and Development, U.S. Environmental Protection Agency, Corvallis, OR, USA

Supplemental Tables and Figures

Table S1. Method summaries for input datasets in this analysis. HUC-8 level estimates were extracted from [*Sabo et al.*, 2019; *Sabo et al., 2021*].

Human N and P Demand	2000 and 2010 U.S. Census block population estimate multiplied by N, P food consumption constants and non-food consumption constant	[*Falcone*, 2016]; [*Sabo et al.*, 2019]
Farm N and P Fertilizer	Annual, county level estimates of farm fertilizer inputs based on a combination of state/county sales data and county level farm chemical expenditure data from Census of Agriculture	[*Brakebill and Gronberg*, 2017]
Cropland Biological N Fixation	Yield based estimate of harvested N content of various leguminous crops	[*Fixen et al.*, 2012]
Harvested Crop N and P Removal	Annual crop N and P revomal values were calculated by reported crop yields by crop nutrient removal coefficients	[*Fixen et al.*, 2012]
Livestock N and P Feed Demand	Multiplied a livestock specific net N and P accumulation constant to Census of Agriculture year end livestock populations	[*Boyer et al.*, 2002; *Han et al.*, 2011]
Livestock N and P Production	Multiplied a livestock specific N and P demand constant to Census of Agriculture year end livestock populations	[*Boyer et al.*, 2002; *Han et al.*, 2011]
Non-farm N and P Fertilizer	County-level estimates of nonfarm fertilizer N inputs based on fertilizer sales data and effective population size	[*Brakebill and Gronberg*, 2017]
Total Atmospheric NO_x-N Deposition	Combined estimates of observed and modeled wet and dry deposition rates	[*Schwede and Lear*, 2014]
Point Source N and P Loads	Multiplying reported discharges by a combination of reported and assumed effluent concentrations based on facility type and treatment level from information primarily reported in the Clean Water Needs Survey	[*Ivahnenko*, 2017]

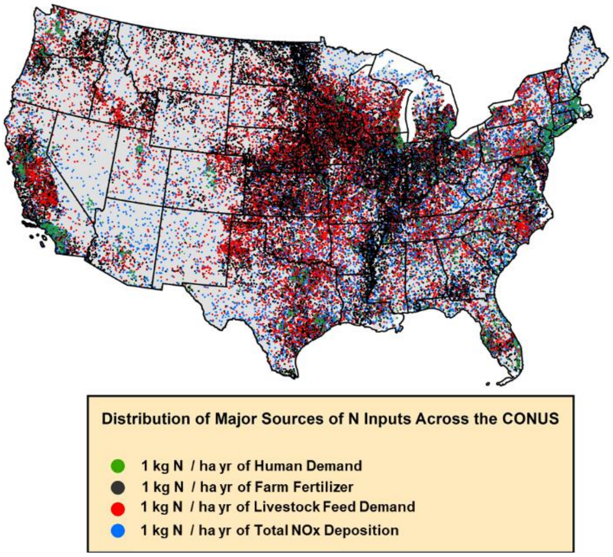

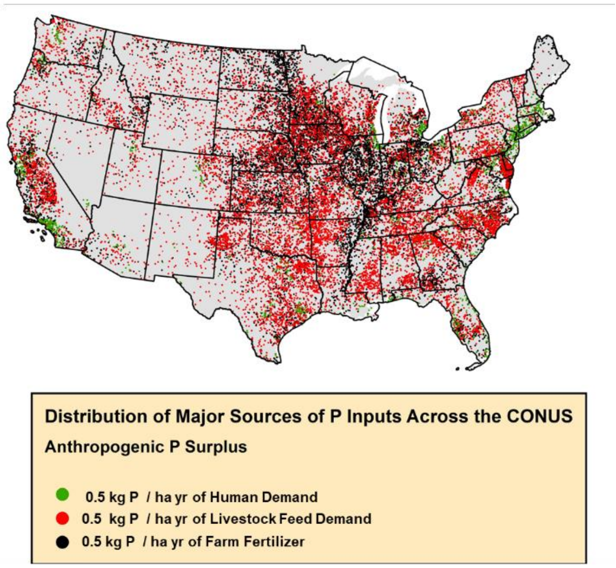

Figure S1. Distribution of some major N and P sources across the contiguous United States. Values are based on an average of input values for 2002, 2007, and 2012 nutrient inventories [*Sabo et al.*, 2019].

References

Boyer, E. W., C. L. Goodale, N. A. Jaworski, and R. W. Howarth (2002), Anthropogenic nitrogen sources and relationships to riverine nitrogen export in the northeastern USA, in *The Nitrogen Cycle at Regional to Global Scales*, edited, pp. 137-169, Springer.

Brakebill, J., and J. Gronberg (2017), County-Level Estimates of Nitrogen and Phosphorus from Commercial Fertilizer for the Conterminous United States, 1987-2012, *US Geological Survey data release, Available at: https://doi. org/10.5066/F7H41PKX, last access, 19*.

Falcone, J. (2016), US block-level population density rasters for 1990, 2000, and 2010.

Fixen, P. E., R. Williams, and Q. B. Rund (2012), NUGIS: A nutrient use geographic information system for the US, *International Plant Nutrition Institute, Brookings, SD*.

Han, H., N. Bosch, and J. D. Allan (2011), Spatial and temporal variation in phosphorus budgets for 24 watersheds in the Lake Erie and Lake Michigan basins, *Biogeochemistry*, *102*(1-3), 45-58.

Ivahnenko, T. I. (2017), Evaluation and use of US Environmental Protection Agency Clean Watersheds Needs Survey data to quantify nutrient loads to surface water, 1978–2012*Rep. 2328-0328*, US Geological Survey.

Sabo, R. D., C. M. Clark, J. Bash, D. Sobota, E. Cooter, J. P. Dobrowolski, B. Z. Houlton, A. Rea, D. Schwede, and S. L. Morford (2019), Decadal Shift in Nitrogen Inputs and Fluxes Across the Contiguous United States: 2002–2012, *Journal of Geophysical Research: Biogeosciences*, *124*(10), 3104-3124.

Sabo, R. D., et al. (2021), Phosphorus inventory for the conterminous United States (2002–2012), *Journal of Geophysical Research: Biogeosciences*, *126*(e2020JG005684), doi:https://doi.org/10.1029/2020JG005684.

Schwede, D. B., and G. G. Lear (2014), A novel hybrid approach for estimating total deposition in the United States, *Atmospheric Environment*, *92*, 207-220.
